# Public health policy in a time of change and disaster in South Africa: 1910–1920

**DOI:** 10.4102/jamba.v8i1.215

**Published:** 2016-09-29

**Authors:** Edwin G. Bain, Jan Venter

**Affiliations:** 1 Bench Marks Centre for Corporate Social Responsibility, North-West University, Potchefstroom Campus, South Africa; 2 School of Social and Government Studies, North-West University, Potchefstroom Campus, South Africa

## Abstract

With the establishment of the Union of South Africa in 1910, the central focus of the newly appointed government was to alter and consolidate the policies of the pre-Union colonies that differed materially in many respects and to substitute them with uniform policies that had to be implemented as a consolidated whole for the Union. This central focus was applied to a number of policies, notably those for the black people, immigration, education, labour, national defence and the development and implementation of railway, mining and agricultural policies. However, an omission occurred with regard to the consideration of a comprehensive public health policy by the political parties and the Union Parliament, consisting of white people only. This article examines this omission during the first 10 years of the Union of South Africa (1910–1920), during the three 5-yearly general elections (on 15 September 1910, 20 October 1915 and 10 March 1920), and argues that this lack of consideration of a comprehensive public health policy can be found in the theory of party political responsible government during unification, which was further developed by Kavanagh, that party political manifestos act as the guiding force behind the policy matters that are discussed and decided upon in Parliament. The article confirms that the reason for not establishing a comprehensive public health policy prior to the outbreak of the influenza epidemic in 1918 was the incidental and piecemeal fashion in which expressions on public health appeared in the published party political manifestos, which in turn influenced the proceedings of Parliament. This political negligence was, however, quickly overturned by Parliament immediately after the epidemic, showing the influence of this demographic disaster on political thinking and action.

## Introduction

The period prior to and after unification in 1910 in South Africa can be traced to the pre-Union and Union policies:

The roots of a dysfunctional health system and the collision of the epidemics of communicable and non-communicable diseases in South Africa can be found in policies from periods of the country’s history, from colonial subjugation, apartheid dispossession, to the post-apartheid period. (Coovadia *et al*. 2009:817)

With the amalgamation of the four British colonial governments in South Africa, namely the Cape of Good Hope, the Orange River Colony, the Transvaal and Natal in 1910, the central focus of the newly appointed government was to alter and consolidate the policies of the pre-Union colonies that differed materially in many important respects and to substitute them with uniform policies that had to be implemented as a consolidated whole for the Union of South Africa. However, an omission occurred with regard to the consideration of a consolidated public health policy by the white political parties, the white Parliament, and the white government in South Africa. The question arises: How does this relate to the statement that the fragmentation of public health history reflects the fragmentation of social history in general? (Fee 1993:xxxix).

This article examines this fragmentation and omission of a consolidated public health policy during the first 10 years of the Union on the basis that a comprehensive national public health policy was not reflected in the published election manifestos of those political parties that participated in the first three general elections on 15 September 1910, 20 October 1915 and 10 March 1920 and, therefore, not on the national agenda of the newly established Union Parliament in terms of the theory of party political responsible government. This examination is conducted by drawing from the published election manifestos of the political parties which consisted of white people only – black people were not directly enfranchised – and their influence in the Union Parliament as a political policy-making body. It further shows that the Parliament, with one exception, in its debates in *Hansards* during this time also did not prioritise a national consolidated public health policy on its agenda. It was only after the great social affliction of the influenza epidemic in 1918, in which an estimated 300 000–500 000 people of all races and cultures died, that a consolidated public health policy in the form of the Public Health Act, 1919b (Act No 36), was adopted for the Union. The authors argue that this lack of an integrated public health policy can be found in the theory of party political responsible government (Kavanagh 1981; Kleynhans 1987; Marais 1989), where party political manifestos act as the guiding force behind the policy matters that are discussed and decided upon in Parliament and on which feedback is given at future elections. The article is an application of this theory, with reference to a particular policy, namely public health policy.

The article concludes that the reason for not establishing a consolidated public health policy was the incidental fashion in which expressions on public health appeared in the published party political manifestos. This, in turn, influenced the proceedings of Parliament, which considered public health matters from 1910 to 1919 in a fragmentary fashion – as supported by the theory of party political responsible government. It further concludes that the manifestation of this theory can be regarded as both positively efficient and negatively efficient with regard to a comprehensive public health policy for the newly established Union of South Africa; positively efficient in the sense that only those national public policy matters that were reflected in the manifestos were followed through in the Union Parliament, and negatively efficient by virtue of the fact that public health policy did not figure as a national priority within the ambit of the manifestos, and therefore also not within the ambit of the business of Parliament.

## Methodology

The methodology followed in this article is historical research, where sources that have recorded past happenings are located and evaluated and then synthesised and interpreted with a view to suggesting causal explanations for events and practices (Welman, Kruger & Mitchell 2005:24). The main theme of historical research concerns the investigation and evaluation of specific events that took place – in the case of this article, the expressions on public health by the enfranchised political parties that took part in the three general elections on 15 September 1910, 20 October 1915 and 10 March 1920 – with the purpose of attempting to find new explanations for, or interpretations of, existing information (Welman *et al*. 2005:24).

For the purposes of this article, the primary sources consisted of the published election manifestos, the programmes and the published public speeches of those white political parties that participated in general elections in South Africa from 1910 to 1920. As there is a direct political connection between political parties’ election manifestos and their legislative programmes (Kleynhans 1987:14), proceedings of the Union Parliament contained in *Hansards* were also consulted to establish the policy expressions, or the lack thereof, on public health by the Union’s intra-parliamentary legislative programme during the 5 years that followed each general election. These primary sources were supported by secondary sources, such as books and articles.

The time frame (1910–1920) has been selected on the basis that responsible party political government commenced with unification in 1910 and was abandoned only after the publication of the Republic of South Africa Constitution Act, 1983 (Act No 110) (Marais 1989:55).

## Theoretical background

The time frame of the research occurred within the theory of responsible party government or Cabinet responsibility, namely a constitutional system which is a parliamentary or Cabinet system of the government. In such a system the Cabinet, or the Executive as it is sometimes called, is part of the legislative institution; therefore, the members of the Cabinet are members of the Parliament (Marais 1989:55). The Union of South Africa had a responsible government system similar to the British practice, as constitutionally provided for by the South Africa Act, 1909, and approved by the British sovereign for South Africa. ‘The election manifesto with its mandatory, binding effect on both the extra- and intra-parliamentary operations of political parties, is basic to responsible government in … South Africa’ (Kleynhans 1987:14).

Referring to the British practice of responsible government, Kavanagh (1981) explains that it postulates a set of relationships between the party, its manifesto and those who voted for it. In summary, Kavanagh (1981) explains the party political manifesto theory as follows:

Policy is made by the party membership, the resulting manifesto is approved, implicitly or explicitly, by voters who support the party, the manifesto pledges set the agenda for legislation in parliament. (p. 15)

In its conception of responsible party government, a party sees its victory in an election as the appropriate way of fulfilling its true aim: the conversion of its platform, programme and principles into actual public policy. The leaders (elected members) of the majority party in the legislative body (for the purposes of this article, the Union Parliament) introduce *Bills* embodying aspects of the party’s programme or manifesto. Then, they are loyally supported by the party members in Parliament, who vote in their favour. Once the legislation is passed, the party leaders make sure that it is faithfully enforced by the administrative departments. At the very next election, the electorate will pass judgement on the party, showing its approval by electing it for a further term, or its disapproval by unseating it (Kleynhans 1987:15).

The party political influence through election manifestos and the proceedings of the Union Parliament, (captured in *Hansards*), in other words how this political system ‘structure the possibilities for healthy or unhealthy lives’ (Fee 1993:xxxviii) on public health cannot be described unless the most important component of political life – the franchise – in South Africa is briefly factored into the discussion, and unless background is provided to the political dispensation in the Union immediately before and after 1910.

## Contextualisation: Franchise and public health

With the process of unfolding political events towards unification, where women of all races had no franchise and where the Cape Province wanted to retain its liberal, non-racial franchise, the northern provinces (the Orange Free State and Transvaal), however, were not to be appeased to extend the Cape franchise to their territories. The result was that ‘… the Union of South Africa started life in 1910 with an illiberal constitution and no direct representation in Parliament for the Africans and Coloureds …’ (De Villiers 1976:141). In essence, the Union of South Africa’s history was permeated with discrimination based on race and gender (Coovadia *et al*. 2009).

Neither the National Convention, who met to consider unification (1908–1909) (attended by white people only), nor the election manifestos of the political parties consisting of white people that contested the first three general elections on 15 September 1910, 20 October 1915 and 10 March 1920, made reference to a consolidated public health policy. These, notwithstanding the fact that prior to 1910, conditions with regard to public health were poor. During the Anglo-Boer War, for example, ‘… medical conditions were primitive, and dysentery killed more British troops than Boer bullets’ (Lewis & Foy 1971:n.p.), and ‘… many more persons were killed by the germs of typhoid than by the guns of the combatants’ (Cluver 1949:317). Only incidental references were made to public health in the published election manifestos of the political parties. It was not until the outbreak of the influenza epidemic in 1918 that the Union Parliament focused on a comprehensive public health policy.

## National Convention and the *South Africa Act*, 1909

The National Convention, which discussed the proposed unification, only made incidental reference to the health of the inhabitants of the country whose political future it was deciding (Cluver 1949:317). In the Minutes of Proceedings of the Convention, the only reference to health was the motion, as amended, put and agreed to, namely ‘… the establishment, maintenance and management of hospitals and charitable institutions’ (Bain 1992; Minutes of Proceedings 1911:81). This agreed-to motion was later adopted in the *South Africa Act*, 1909, as one of the functions of the provincial councils that had to be established (section 85[v]). Elementary education (i.e. education other than higher education) (section 84[iii]) was also assigned to the four provincial councils, which had been interpreted as school medical inspection and hygiene services (Bain 1992; Government of South Africa 1924:184).

The term ‘public health’ was not mentioned in the *South Africa Act*, 1909. It was merely considered to be a matter under the control of the Union government in the sense that, after the constitution of the Union, the implementation of public health matters existing in the four provinces in terms of their discordant policies would be overseen by the Department of the Interior, with an Advisory Medical Officer of Health for the Union in Pretoria (currently Tshwane), and three Assistant Health Officers, with headquarters in Cape Town, Durban (currently eThekwini) and Bloemfontein (currently Mangaung), respectively (Bain 1992; Government of South Africa 1921:232).

Eight years after unification, no steps had been taken by the Union Parliament towards creating a central Department of Health. Also, the functions assigned to the Department of Health, which came into being in 1920, had never been properly defined by the legislature (Bain 1992; Report 1919:12).

In terms of the *South Africa Act*, 1909, the legislative authority of the Union was vested in the Parliament of the Union, which consisted of the (British) sovereign, the Senate and a House of Assembly. Up to the date of the first general election on 15 September 1910 (three and a half months after becoming the Union on 31 May 1910), no legislature, as envisaged in the *South Africa Act*, 1909, existed in the Union. During this time, the governing body of the country was vested in the sovereign and was administered by a Governor-General appointed by the sovereign as his representative (sections 8, 9) (Bain 1992).

The Governor-General’s first task was to appoint a Prime Minister to govern the Union. The British government was of the opinion that Louis Botha, at the time the head of the Transvaal government, was to be appointed as the Prime Minister of the Union (Bain 1992). He became the country’s leading political figure and *de facto* Chief Executive from 1910 to 1919, with powers similar to those of his British counterpart. Botha envisaged unity between English- and Afrikaans-speaking South Africans through his politics of reconciliation. After Botha’s appointment, he selected his Cabinet members from the ruling parties in the four colonial governments. In practice, he was the leader of the majority party or coalition in the House of Assembly (Marais 1989:15).

The stage was now set for the political parties to enter the Union’s political domain through their party political manifestos and their members’ participation in Parliament on the basis of these manifestos.

## Manifestos and debates in *Hansards* on public health policy

Public health as a national priority was conspicuous in its absence when the newly appointed Prime Minister gave notice to the House of Assembly of a number of *Bills* of priority to carry unification into practice (Bain 1992; Government of South Africa 1910).

The manifestos of the political parties that took part in the general elections from 1910 to 1920 also reflected a lack of concern for, as well as incidental references to, public health matters in the newly established Union.

### The 1910 general election

An analysis of the election manifestos of three of the political parties that took part in the 15 September 1910 general election (four parties took part, but a manifesto could not be traced for one of them, the Independents) (Kleynhans 1987:21) give an indication of the importance, or not, of the future state of the health of the people and the way in which each party sought to deal with it if they won the election and became the ruling party in the legislature.

The *De Zuid-Afrikaanse Nationale Partij* (ZAP) of Louis Botha, which won the election with 67 seats, stated 11 objectives and principles in its election manifesto which the party desired to implement in the Union. The second of these objectives and principles dealt with how the party envisaged its involvement in ‘a healthy South African spirit in dealing with our political and national problems’ (Programme, 1910:n.p.). No mention was made of the other components of a comprehensive health plan for the country, namely the physical and social.^1^ The other national objectives dealt with the black people question, immigration, education, labour, national defence, and the development and implementation of railway, mining and agricultural policies. As far as railway, mining, and agricultural policies were concerned, these objectives were indeed dealt with when the party formed a government after the first general election in 1910 (Government of South Africa 1910:*passim*).

The programme of the contesting Unionist Party of South Africa (it won 39 seats, making it the official pro-imperial opposition party between 1910 and 1920) dealt with the following matters that were of concern to them, namely, an impartial public service, education, black people policy, excise, agriculture, and industrial development (Programme 1910). As for health matters, the Unionist Party of South Africa merely referred to the ‘… introduction of legislation where necessary (and particularly in cases where the nature of the occupation may have any injurious effect on health)’ (Programme 1910:24).

In the case of the South African Labour Party (it won four seats), proposals were made relating to education, labour, women rights (no women had voting rights at the time), defence, mining, agriculture, native policy and Asiatics (Manifesto 1910). The Party only referred to ‘… free medical inspection and treatment of school children’, ‘… compensation to all workers for industrial diseases’ and ‘… proper safeguards for the health of workers in mines and factories’ (Manifesto 1910:27). The South African Labour Party therefore also made no mention of how it proposed to deal comprehensively with the health issues of the country.

Thus, matters relating to a comprehensive public health policy and the state of the health of inhabitants of the newly formed Union received little attention from the political parties that contested the first general election, and was not a national priority for the parties referred to. An uncertain situation therefore existed as how to deal comprehensively with the country’s health issues as the political parties sought to deal with the other (what they considered more important) national policy matters of the Union.

During the proceedings of the First Parliament of the Union of South Africa, the Governor-General in his opening speech to both Houses of Parliament on 04 November 1910 emphasised that the policies of the pre-Union colonies differed materially in many important respects and that, sooner or later, it would be necessary, by alteration and consolidation, to substitute uniform policies that had to be implemented in the whole of the Union. Among the measures that he felt were to be submitted to the Houses for the continuation of the implementation of services in the Union were *Bills* dealing with estimates of revenue and expenditure, the audit, naturalisation, railways and harbours, posts and telegraphs, immigration, and stock and plant diseases (Government of South Africa 1910:Cols. 19,20). No mention was made of the diseases that affected human beings (Bain 1992).

The only inference that can be made from the above observations is that the Union legislature, after its constitution, was not considered by the Governor-General and the political parties to deal with human health matters as a national priority; thus, it would continue with the implementation of pre-Union health policies suited to the individual colonial governments.

Furthermore, the creation of a separate portfolio of public health for the implementation of public health policies was not considered a priority. On being asked in the House of Assembly whether the government would advise the appointment of a minister to hold the portfolio of public health, the Minister of Interior (who was also responsible for health matters) replied that it was unnecessary as matters of public health could be dealt adequately by the Department of Interior (Government of South Africa 1910:Col 30).

During the years 1910–1911, March 1911 (12 months after the formal opening of Parliament by Proclamation on March 31, 1910) can be considered as important in the development of a national health service because it was the first attempt by the Union legislature to embark on the adoption of a *Public Health Acts Amendment Bill*, 1911,^2^ to serve the interests of the country. On the Minister of Interior’s moving of the Second Reading of the *Bill*, he stated that it was a short and simple *Bill* which contained only two major provisions, namely, one providing for the appointment of an Officer of Health for the Union, and the other providing for certain measures that might become necessary in the event of a major outbreak of an epidemic in the country. The details of these measures were not mentioned by the Minister. Also, no mention was made of the establishment of a separate ministerial portfolio for public health (Bain 1992; Government of South Africa 1910:Col. 1724).

On the grounds of various objections to the *Bill*, namely, that it was not a well-thought-out piece of legislation, that it was an apology for a *Health Bill*, that animal diseases were catered for far more extensively than for diseases of human beings, and that the introduction of it was crude and superfluous and had not taken proper cognisance of the health circumstances at the time, the *Bill* was negatived^3^ at the Second Reading and did not reach the Committee stage (Government of South Africa 1910:Cols. 1725, 1726, 1729, 1731, 2942). Thus, the Minister of Interior’s vision of making provision for certain measures in the case of an epidemic was not considered important by the legislature, for the reasons cited.

During the years 1911 to 1913, the most important enactments dealt with were agriculture, (*Hansard* 1910–1911:Cols. 968, 989, 995), defence (Government of South Africa 1912:Cols 619, 659, 741, 755), railways (*Hansard* 1910–1911:Cols.2831, 2843, 2845) and black people’s land ownership (Bain 1992; Government of South Africa 1913:Cols. 2270, 2439, 4482, 2530, 2825).

A comprehensive consolidating and amending *Public Health Bill*, 1913, designed to replace the separate health acts of the provinces was drafted in 1913. The draft *Public Health Bill*, 1913, was submitted to local authorities for their information and comments in the following year (1914), but owing to the First World War (1914–1919), it was not proceeded with. Also, as a result of the vagueness of the *South Africa Act* 1909, concerning public health matters, the four provincial administrations proceeded with their individual provisions of health services, which was divergent in certain respects (Bain 1992; Government of South Africa 1924:184). For example, concerning the registration of births and deaths, a different series of laws applied in each of the provinces. It was only recognised at the time of the influenza epidemic (1918) that it was desirable to introduce a uniform system of registration throughout the Union, but it was not accomplished at the time. Although the registration of births and deaths of white people could have been regarded as reliable throughout the Union, the situation among black people was ‘… a matter of great difficulty’ (Bain 1992; Government of South Africa 1921:175).

The manifestos of the political parties that contested the 15 September 1910 general election, therefore, corresponded with the component parts of the theory of responsible party government by only relating to the policy matters of the party political manifestos, as adopted by the political parties. These manifestos offered no clear indication of how the parties sought to deal with the future health issues of the country at a national level. In the proceedings of the Union Parliament, the draft *Public Health Bill*, 1913, was an exceptional move and was introduced outside the scope of the manifestos. One can speculate whether this would have been pursued to its logical finalisation, were it not for the First World War (1914–1919).

### The 1915 general election

The political parties participating in the October 1915 general election also followed the theory of party political responsible government in the sense that their manifestos and programmes, firstly, responded to the white male electorate on their achievements on the undertakings in their previous election manifestos, and, secondly, how they had attained these undertakings or not, followed by their undertakings on how to deal with the existing and future challenges. As will be shown subsequently, these future challenges did not include public health as a national priority.

In the run-up to the October 1915 general election, which was contested by the *De Zuid-Afrikaanse Nationale Partij* (it won 54 seats), the Unionist Party (it won 40 seats), the National Party (it won 27 seats), and the South African Labour Party (it won four seats), public health was not mentioned by any of the parties in their election manifestos on their reports back to the electorate. The achievements and objectives of the political parties focused on jingoism^4^ (*Programma van Aktie van de Nationale Partij van de Kaap Provinsie*), the amnesty of political detainees, defence, agriculture, industries, the poor white people’s issue, matters concerning Asian and black people, martial law, education, black people’s labour issues, language issues, financing and provincial councils and local authorities (*Redevoeringen, Speciale Kongres van de ZAP*; *Manifest en Programma van Aktie, Nationale Partij van de Kaap Provincie*; *Volledig Verslag van het Kongres van de Nationale Partij van Transvaal; Die Hertzogroesprake, Deel 3; Manifes van die Arbeids Partij*; Speeches delivered by General L. Botha and Ministers at *De Zuid Afrikaanse Partij Special Congress at Bloemfontein; Manifesto, the National Party of the Cape Province*) (Bain 1992).

Three years after the 1915 general election, an influenza epidemic broke out in the Union, which forced the Botha government and the political parties to prioritise the health of the nation as a matter of urgency: a realisation of the importance of a consolidated health policy for the Union that was hitherto not accorded its rightful place in the political arena of the newly established Union. This epidemic of September 1918 was South Africa’s worst demographic disaster. It formed part of an international influenza pandemic, also referred to as the ‘Spanish Flu’, and a month into its devastating death toll, as ‘Black October’ (Philips 1988:63). The epidemic, in no uncertain terms, emphasised the importance of a national consolidated health policy for the country in the place of incidental, and often differing, health legislation that was still intact from the pre-Union political era.

### Health policy and the influenza epidemic, 1918

The fact that the state of health of the inhabitants of a country can be as important to the future of the country as the state of its political and economic development was forcibly brought home to the legislators of the Union by this single, crucial health disaster in 1918.

For the inhabitants of the country of all races and cultures (infectious diseases show no respect for constitutional boundaries and/or racial, gender and cultural differences), the aftermath of this epidemic was devastating. The inhabitants of the Union were in a recovery phase after the Anglo-Boer War and its concentration camps (1899–1902) – the war directly and/or indirectly affected not only the members of the two warring parties (the Boers and the British) but all of the country’s inhabitants. Then followed the aftershock of the 1913 opening of the National Women’s Memorial, commemorating women and children who died in the British concentration camps; the 1913 miners’ strikes and riots on the Witwatersrand, which continued into 1914, resulting in martial law being proclaimed; the First World War (1914–1919); and the outbreak and suppression of the Rebellion in 1914 (Government of South Africa 1921:232). The outbreak of the influenza epidemic, and the severe and overwhelming shock and grief that followed in its wake, could not have come at a more inopportune time for all the inhabitants of the Union.

The ravages of the epidemic were felt far and wide throughout the Union, with the official total death rate of 22.80 per 1000 of the population (Report 1919:23), totalling 139 471 deaths (Report 1919:par 49). This figure, however, was not regarded as a reliable expression of the number of deaths and it had been suggested that ‘… perhaps a figure of 300 000 would be a reasonable estimate’ (Philips 1988:63), while another source estimated the death toll at 500 000 (Bain 1992; Health24 2015).

Soon after the outbreak began in September 1918, there met in Bloemfontein, under the chairmanship of the Minister of Interior, white people’s representatives of the central government and the four provincial governments, of the four municipal associations, of the Cape Divisional Council Association, of the Transvaal Chamber of Mines, of the Native Labour Recruiting Associations, and of many medical and social organisations. This is an indication of the importance of mainly whites-only people’s interest groups to the governmental policy-making process, albeit of an *ad hoc* nature, caused by the influenza epidemic. During that occasion, namely the Public Health Conference, the representatives considered and made recommendations on steps that had to be taken to remedy the flaws in the existing legislation, so as to place public health on a sound footing (Cluver 1949:317). It was therefore a combined effort from the public and political interest groups to plan for, and avert a similar health disaster in future. These recommendations conceded that there had been a lack of political and governmental action in attending to a national health policy since unification 8 years previously. The result of the recommendations was the expeditious adoption, a year later, of the *Public Health Act*, 1919b (Act No 36), driven by the political parties in Parliament.

### Expeditious health legislation reform

The legislative process with the adoption of the *Public Health Act*, 1919b (Act No 36), was unusual in the sense that the *Public Health Bill*, 1919, passed speedily (within 6 months) through all the stages of law-making by the legislature during that year, compared to the previous and subsequent attempts at passing health legislation. The First Reading was on 22 January 1919, the Second Reading on 05 February 1919, the Committee stage on 25, 28 and 29 April 1919, and on 02, 05, 07 and 08 May 1919. Amendments were considered on 12 May 1919, the Third Reading was on 19 May 1919, it was amended by Senate on 09 June 1919. Amendments were considered on 10 June 1919, and it was assented to by the Union Parliament on 20 June 1919 (Government of South Africa 1919a:xiv). During the Second Reading debate on this *Bill*, the Minister of Interior stated that the new and salient features contained in it were the creation of a central health administration to control and coordinate all health duties in the Union (Government of South Africa 1919a:23).

As an example of the tedious legislative processes that usually accompany the adoption of health legislation, the following can be referred to: attempts to consolidate laws relating to medical practitioners and related professions were before Parliament for more than a decade after 1918, before the *Medical, Dental and Pharmacy Act*, 1928 (Act No 13) was assented to by the Union Parliament.

Although it was necessary to amend the *Public Health Act*, 1919b (Act No 36) at no less than 21 occasions because it was an Act which was born out of emergency and disaster (Government of South Africa 1977:Col. 3137), it served the needs of the inhabitants of the country from 1919 to 1977 (Bain 1992; Departement van Gesondheid 1977, 1978:3).

The state of health of the people of the Union was clearly not an insignificant matter anymore, and what previously was not regarded a national priority, became one after the ravages of the influenza epidemic.

### The 10 March 1920 general election

The manifestos of the political parties that contested the 10 March 1920 general elections once again accorded with the component parts of responsible party government by only relating to the contents of the party political manifestos, as adopted by the parties, by giving no direct indication as to how the parties sought to deal with the future health issues of the country following the disastrous health situation described immediately above. Only the Unionist Party (neither a leading nor opposition party) referred in passing to the *Public Health Act*, 1919b (Act No 36), as being an instrument that its members helped to place on the statute book (Manifesto 1920). Similarly, public health did not figure as a priority during the 1921 general elections, which came about as a result of the indecisiveness of the 1920 election (Bain 1992). The adoption of the *Public Health Act*, 1919b (Act No 36), was seemingly regarded by the political parties as a measure that would meet the needs of the inhabitants of the country for the foreseeable future.

## Conclusion

Immediately prior to unification in South Africa, the National Convention (1908–1909), which was attended by white representatives only, did not consider a national health policy as an important issue for the agenda of the soon to be created Union of South Africa. Even the *South Africa Act*, 1909, the result of the National Convention and the British sovereign’s deliberations, only referred in a fragmentary fashion to curative and personal health, based on the pre-Union Acts – hospitals, charitable institutions, school medical services and hygiene. After unification, the party political manifestos of the white parties that contested the three 5-yearly general elections (on 15 September 1910, 20 October 1915 and 10 March 1920), also referred incidentally to public health. These *ad hoc* references to public health continued in the proceedings of the Union Parliament during the time. The only exception was the submission of a comprehensive *Health Bill*, 1913, which was outside the pledges of the political parties’ 1910 general election manifestos, the importance of which was overtaken by the First World War (1914–1919) that the Union government participated in.

The *Public Health Act*, 1919b (Act No 36), can be considered as a watershed moment for the Union, with regard to health matters, which were dealt with in a comprehensive manner. For the first time since the establishment of the Union in 1910, a consolidating health policy for the inhabitants of the Union was combined in a single act.

This watershed moment was prompted by one event, and one event only, namely the influenza epidemic of 1918 (with an estimated death toll of 300 000–500 000). The fact that it was not prompted by the pledges elucidated in the election manifestos of the political parties that contested two of the three general elections (1910 and 1915) confirms the theory of responsible party political government, by the practice that political parties stayed within the ambit of the expressions in their election manifestos and, thus, responsibility to the party decisions. The manifestos only referred to public health in a piecemeal fashion, and so did the discussions in Parliament, by not diverting from the expressions in the manifestos as per the dictates of responsible party government, except for a brief once-off moment in 1913, and later a fully-fledged parliamentary process in 1919, as a result of the influenza epidemic.

Responsible party political government therefore can be regarded as positively efficient only to those national interests expressed in the election manifestos of the white male political parties of the Union during the period 1910–1920. Public health was not one of these national interests. As shown, party political election manifestos could also be negatively efficient as they related to the process of dealing with the national issue of a comprehensive public health policy for the Union. A comprehensive health policy for the Union was not considered a national priority, having not been earmarked in the pledges of the political parties’ election manifestos. For 9 years after unification, other more important national matters were on the agendas of the political parties that contested the general elections. It was only as a result of the influenza epidemic of 1918 that public health in the Union of South Africa at last became part of the Union legislature’s social development in a consolidating and national sense.

## Footnotes

1 The World Health Organization (1977) defines ‘health’ as the complete physical, social, and spiritual wellbeing of people.

2 ‘Acts’ in the title referred to pre-Union Acts.

3 Disagreement with a *Bill* by rejecting the motions for second or third readings. This also meant the rejection of the *Bill*.

4 ‘Jingoism’ as specifically referred to in the *Manifest en Programma van Aktie van de Nationle Partij van de Kaap Provincie*, against the pro-imperial opposition party (the Unionist Party) between 1910 and 1920. In general, it refers to any ‘uitlanders’ (outsiders) with imperial annexation in mind: ‘a policy … which appeals most strongly to the instincts of self-interest and to the illegal appropriation of other people’s property’ (http://www.angloboerwar.com/books/30-reitz-a-century-of-wrong/715-reitz-capitalistic-jingoism-first-period).
